# VEPdgets: Towards Richer Interaction Elements Based on Visually Evoked Potentials †

**DOI:** 10.3390/s23229127

**Published:** 2023-11-12

**Authors:** Philipp Wolf, Timo Götzelmann

**Affiliations:** Nuremberg Institute of Technology, Chair of Ambient Intelligence, D-90489 Nuremberg, Germany

**Keywords:** brain–computer interface, BCI, VEP, interaction, HCI, interaction elements, control elements, controls, widgets, VEP-widget

## Abstract

For brain–computer interfaces, a variety of technologies and applications already exist. However, current approaches use visual evoked potentials (VEP) only as action triggers or in combination with other input technologies. This paper shows that the losing visually evoked potentials after looking away from a stimulus is a reliable temporal parameter. The associated latency can be used to control time-varying variables using the VEP. In this context, we introduced VEP interaction elements (VEP widgets) for a value input of numbers, which can be applied in various ways and is purely based on VEP technology. We carried out a user study in a desktop as well as in a virtual reality setting. The results for both settings showed that the temporal control approach using latency correction could be applied to the input of values using the proposed VEP widgets. Even though value input is not very accurate under untrained conditions, users could input numerical values. Our concept of applying latency correction to VEP widgets is not limited to the input of numbers.

## 1. Introduction

Various types of brain–computer interfaces (BCIs) are already present in the mass market. With them, a promising new input modality is available alongside standard input devices such as keyboard, mouse, gesture control or gaze tracking, offering users a completely new way of interacting with their environment. Originally from the medical application area, BCI are currently shifting to the consumer area and thus becoming accessible to a larger group of people.

### 1.1. Brain–Computer Interfaces

A brain–computer interface (BCI) allows the measurement and interpretation of the brain’s neural activity using electroencephalography (EEG), providing a direct interface between the brain and a computer. Users can thus interact with the computer without moving their extremities.

A distinction is made here between three signal acquisition categories. In invasive interfaces, data are obtained by placing microelectrodes within the meninges of the brain. In semi-invasive methods, the electrodes are applied only under the scalp but not inside the skull. Entirely without surgery, noninvasive techniques can be achieved by contact of the electrodes with the scalp [1,2]. Non-invasive devices often resemble a headset attached to the head, similar to a cap or a headband. The signals needed for analysis are sampled using electroencephalography (EEG). The advantages of this already established data acquisition method lie in the high temporal resolution as well as in simple and low-risk applicability [3] at comparatively low costs [2] for non-invasive systems. The brain signals required for the analysis are generated either by external stimuli, so-called stimulation, or by the user’s own power and imagination and thus represent the intention of a person using the system and are interpreted accordingly, resulting from the accomplishment of a task [3].

Differentiated, these human–machine interactions are considered active, reactive, and passively occurring signals [4]. In the case of active signal generation, the output is derived from brain activities that occur independently of external events directly and consciously controlled by the person using them. BCIs belonging to the active category are based on Motor Imagery (MI) and require a particularly large number of training sessions as well as calibration data to enable effective deployment on the day of use [5]. In addition, these generally suffer from low information transfer rates and unreliable classification accuracy. Reactive brain activity is understood to be brain activity that is evoked by external stimulation and can be clearly traced to a trigger. These waveforms in the electroencephalogram, related to an observable event, are also called event-related potentials (ERP). Passive generation derives its outputs from random brain activity that occurs without the purpose of voluntary control or external stimulation to implicitly gain information about the actual state of the person using it [2,4].

Several limitations also accompany the use of BCI systems. In terms of its operation and design, some types of BCI cause fatigue on the user’s part due to uncomfortable operation over long periods. Also, BCI deficiency, also known as BCI illiteracy, in which the user is unable to control a type of BCI in an appropriate manner, is a problem [6].

### 1.2. Visually Evoked Potentials

A reactive paradigm often invoked for brain–computer interfaces and used for this work represents the Visual Evoked Potential (VEP) or Visual Evoked Reaction. Electrophysiological analysis of the VEP is considered a powerful tool for studying visual function, improving reliability in detecting visual disorders [7]. This measurable potential occurs in the visual cortex after a visual stimulus is viewed. Hereafter, visual stimuli consist of a binary sequence of luminance changes. For this purpose, it is sufficient to direct attention or gaze to a stimulus. A flash or flicker of a light source generates a reaction in the visual cortex located at the back of the head after a temporal offset. The further the stimulus comes into visual focus, the stronger the amplitude of the response potential, and the easier its detection [8]. Features such as color (Chromatic VEP), shape, luminance, and intensity influence potential response [9].

Studies from 2017 in [10] found that using a VEP-based BCI, even under mental stress, requires little attention from the person using it to achieve optimal accuracy, as it does not significantly impair auditory attention or working memory. The flickering stimuli, however, can distract from completing the task at hand and can also cause eye fatigue [4,11,12].

Repetitive visual stimulus, or RVS (Repetitive Visual Stimulus) of VEP, is divided into several categories in terms of its frequency. In Transient VEP (TVEP), the visual stimuli are emitted at large and discontinuous intervals from each other. If the stimulation rate is sufficiently low, the response may be completed before the next visual stimulus arrives. The potential response is then referred to as a transient and non-persistent evoked response. In solid-state VEP (SSVEP), the visual stimuli are emitted at equal intervals from each other. The potential response in the cortex is the same as that of the steady frequency of the light source. Multi-Frequency VEP (MFVEP) is characterized by the visual stimuli appearing based on multiple SSVEP frequencies overlapping simultaneously. The combinations of the few frequencies with and among each other result in a higher number of distinct and distinguishable stimulus patterns. In Code-Modulated VEP (CVEP), the visual stimuli are emitted based on encoding with differently delayed versions of a pseudorandom stimulus sequence [13].

The VEP paradigm is considered a promising brain–computer interface in many application areas. Still, so far, it is only used as a switch to trigger action from its moment of recognition, thus allowing only very coarse and time-delayed inputs. This paper presents a new VEP-based interaction technique that moves beyond the related work described in the next section.

## 2. Related Work

Originating from the medical field, a wide variety of applications have already been realized under the VEP paradigm, such as video games, control of wheelchairs and robots, and many more. A corresponding overview paper on AI algorithms for VEP and P300 signals for use in motor rehabilitation applications can be found in [14]. The majority of all studies in the field of brain–computer interfaces using VEP mainly deal with the evaluation of systems in terms of information transfer rate, classification accuracy, test success rates, and general usability by evaluating diverse applications.

For all the studies described below, with a few exceptions, the authors designed their own systems, which differed in the EEG signal acquisition systems and signal processing in classification, stimulus triggering, and user feedback. All studies used exclusively non-invasive methods for signal acquisition. Within the work considered for this section, the VEP interactions, without exception, function as action triggers for value input in various applications based on them as soon as the BCI recognizes the occurring potential. The studied works (also see [15]) can be divided into those presenting pure approaches, which are used only by using the VEP, and hybrid approaches, using an additional technology to improve or extend the system. Because of its extent, a structured and detailed overview of the analyzed related work of the pure and hybrid approaches is outsourced in the tables of Appendix A.

### 2.1. Hybrid Approaches

Pure VEP approaches have so far been limited in their input capabilities or limited in their reliability. Therefore, there are several hybrid approaches that use VEP together with other technologies to provide enhanced or more accurate VEP interaction. On several occasions, VEP has been used in combination with electrooculography (EOG) (e.g., [16,17,18]). Here, EOG was used to improve the detection of the stimuli. VEP were also used with electromyography (EMG). Here, a glove was used to implement hand closure for navigation of different areas of the screen [19]. In combination with event-related desynchronization (ERD), VEP was used to control a cursor. Here, horizontal control was performed using VEP, and vertical control was performed using ERD. BCIs were also used to link control with VEP and P300, representing an event-related potential, but with different characteristics [20] than those of VEP. Similarly, a BCI was used in part by VEP for the command control of an aircraft simulation [21].

### 2.2. Pure Approaches

In addition to the hybrid approaches, a number of works exist for which input exclusively uses VEP. As with the hybrid approaches, much of the work applies VEP to *navigation* in applications. However, some approaches further allow for dedicated *value input*. Since this is the focus of this paper, these approaches are described in more detail below.

**Navigation.** Many approaches apply VEP to motion implementation in games, simulations, and virtual applications. The stimuli are mostly placed statically in the user’s field of view. Bieger et al. [22] demonstrated the applicability of control in a 2D game. Other approaches allowed controlling 3D environments in a similar context (e.g., [12,23]). In addition, they have been applied to virtual control of cars ([24,25,26]), of robots via HMD [27] as well as of a virtual wheelchair [28]. In addition, there are also approaches that focus on stimuli that are not static but move in a virtual space [29,30]. There are also already approaches for control in reality, such as controlling an actual wheelchair [31] or a quadrocopter [32], as well as for navigation by means of three (left, right, forward) or, in some cases, four options.

**Input of Values.** The use of VEP originated in the medical field. It was often investigated to provide patients unable to communicate conventionally (e.g., Locked-in syndrome) with a channel that allowed them as little interaction with their environment as possible, without much effort. Therefore, the standard use case was a keyboard with alphanumeric values to create a textual bridge. A control similar to those previously described for navigation was presented by Volosyak et al. [33]. Here, the selection of a character could be controlled two-dimensionally via four direction keys, starting from the previously selected one. Including the selection confirmation stimulus, a maximum of five simultaneously active stimuli could be recognized in the field of view to select 32 different values and actions. After each activation, detection was deactivated for two seconds to allow refocusing of the using person. This resulted in large distances on the keyboard as well as a large total input time.

Cheng et al. [34] tested their BCI system with the rudimentary operation of a numeric keypad with special characters. Here, 13 different stimuli that could be activated simultaneously were used in the form of a number pad with values from zero to nine, a delete and confirmation field, and a general stimuli activation element. In the tests, only 8 out of 13 subjects were able to successfully enter and confirm a sequence of numbers. The disadvantage mentioned was the fact that 13 elements were flickering at the same time, which, from the point of view of other studies, was also due to visual fatigue [35].

To gain further insights into the optimal number of stimuli that can be activated simultaneously to counteract fatigue, Li et al. [36] first investigated interaction quality with a single-layer 6 × 6 field matrix with a total of 36 different values, and then a two-layer 2 × 6 field matrix in which the desired value was not directly completed but via an additional input step. The 36 different symbols were divided into six fields of six values. By first group selection, these were again distributed to six fields, resulting in a two-step selection. The results of the tests with ten participants clearly showed that the crowding effect was reduced. The insights gained were that stimuli should not be placed too close to each other, and that accuracy and information transfer rate can be increased with a two-level input.

Another multistage approach [37] divided the Latin alphabet including a space with its 27 symbols into three elements (A–I; J–R; P–Z + space). After their activation, the selection was again divided into three elements, and thus, until after completion of three-step interaction, an explicit symbol could be selected. Including the confirmation and deletion functions, a maximum of five elements were thus evoked simultaneously. Although the use of fewer commands was considered to be a disadvantage, test participants found it easier to select the same command several times in succession. Because the sequence of commands corresponds to the same visual stimulus, there was no delay due to gaze shift. For example, the using person had to aim at the same target for three commands to write the letter ‘A’. This observation can be taken into account when changing the order of letters in the decision tree.

### 2.3. Conclusions

Due to continuous improvements in signal processing, including artificial intelligence, BCIs are becoming more accurate and are also being used for applications outside the medical field [27]. We distinguished between hybrid and pure approaches. The latter approaches use VEP exclusively for navigation in virtual or real environments, or for value input into computers. As it turns out, both navigation and value entry have so far been implemented exclusively by triggering switches. When presenting several simultaneous switches (e.g., for entering characters), it is a common difficulty to display them simultaneously. In order to use them, there are often several successive inputs necessary. In the following section, we present a novel VEP technique based on temporal control. It offers several alternative interaction options for VEP to enter values based on temporal intervals.

## 3. Approach

This conceptual section first defines the boundary conditions for a system using our proposed method and presents the fundamental interaction of the VEP-based interaction elements.

### 3.1. Boundary Conditions

In this section, we first define the hardware requirements for our VEP approach. We then define some constraints based on the papers discussed in Section 2 and the findings from their studies. The brain–computer interface should meet the following requirements for the planned experimental setup:

- Sufficient transmission rate of the system;
- Sufficient accuracy and speed of target detection;
- Sufficient number of selectable visual targets;
- Compact design of the system.

#### 3.1.1. Visual Stimuli

VEP stimuli’s detection rate and accuracy depend on several factors defined in more detail below.

**Simultaneous Number of Stimuli.** A high number of flickering stimuli leads to rapid visual fatigue [36], resulting in an unpleasant user experience. Overall, all the work is considered in Table A1 and Table A2. The entered data were not implemented as single-step alphanumeric value entry. An average of four to five simultaneously active visual stimuli were used in the user’s field of view. This represents the maximum number of visual stimuli to be used.

**Size of Stimuli.** The response detection rate depends primarily on the size of the area of the field of view occupied. The larger the area of the stimulus trigger, the faster a response is achieved; conversely, the smaller the area, the more difficult it is to obtain a response.

**Distance of Stimuli.** Furthermore, a high number of stimuli of constant size leads to stimuli being placed too close together. As already recognized by Li et al. [36], this can lead to a crowding-out effect, reducing recognition quality. A recommendation for the correct spacing between stimuli was given by Ng et al. The spatial separation of the competing visual stimuli should be at least five degrees of visual angle. This is consistent with the the Stiles–Crawford effect, which states that brightness perception is weak at the pupil margin and highest at the point of best vision, near the center of the pupil [38]. The further the distance of a stimulus neighbor, the further it moves out of the focused area.

#### 3.1.2. Value Display

To be able to make inputs correctly, it is necessary that the user receives visual feedback on his interaction at any time.

**Minimization of Input and Output Distance.** During the VEP interaction, in which the eyes are focused on the visual stimulus, a value display for monitoring the concurrent interaction process must be recognizable as information feedback to offer the possibility to terminate the interaction at any desired time. We suppose the value display is too far away from the stimulus. In that case, the focus area switches between the display and the stimulus by changing gaze, which can lead to an undesired stimulus response loss and thus to premature interaction termination. Accordingly, an information display must be located in the same focal area as the stimulus. Still, the display must not interfere with it in its evoking property by covering it over or interfering with it by its own segregation—visual stimuli in the form of digit changes.

**Midas Touch Problem.** Conversely, the Midas touch problem that may arise in the process must be circumvented. With gaze-controlled interfaces, any fixation on an optical interface element can lead to its activation, even if there is no such intention on the user’s part [39]. Such accidental interaction activation should be avoided when merely looking at or checking the value display.

### 3.2. Development of Interaction Technique

In this section, interaction techniques for VEP are presented. These techniques are used for inputting values via desktop and VR interaction using an HMD.

#### 3.2.1. Temporal Interaction Approaches

The use of a temporal component in the interaction with user interfaces is not new and has already been used in a variety of applications. Common examples include Morse codes (encoding long and short signals), triggering alternative input levels on keyboards or touch gestures.

The term temporal interaction or control is often used in the context of temporal data. Hibino and Rundensteiner [40] introduced a temporal visual query language. They used usual GUI interaction elements and temporal diagrams for the navigation and queries of video sequences. Another approach on temporal data by Mathew et al. [41] used spatial and temporal interaction techniques for audio authoring. In their study, they investigated inputs such as physical sliders, touch interaction and a phantom 3D controller. They compared the completion time for tasks to control timeline of audio data. Jeuris et al. [42] presented a further approach based on timeline GUI elements for activity lifecycles of a temporal desktop interface.

The term is also used for instrumental interaction. Beaudouin [43] raised this term first and tried to minimize the temporal offset between interaction and the reaction of the object. Other approaches picked up on this and used eye-tracking for instrumental interaction. Huckauf and Urbina [44] used pie menus to play notes. A dwell time was necessary here to avoid the unintentional triggering of actions (see Section 3.1.2). Another approach EyeHarp [45] used other interaction elements such as sliders, repeat buttons, tabs and switches controlled with gaze input instead of pie menus. Temporal control was, however, used here to omit the Midas touch problem by definition of a minimum dwell time.

#### 3.2.2. Temporal Control for VEP

This section presents the interaction technique of temporal control for VEP, which is developed using latency correction. Since the visually evoked responses can basically only be detected after a certain temporal interval at the back of the head and after a further time interval via signal processing and subsequent classification, the temporal delay duration is calculated for the approach of this interaction technique of VEP responses. An additional latency arises when the visual stimulus is no longer present; for example, when the observer closes their eyes or looks at something else. When knowing these latencies, exact interaction times and gaze dwell times can be determined, which are necessary for a more reliable and accurate interaction. These time intervals are determined by a calibration step consisting of several iterations. In the following, the procedure of latency correction is explained in more detail.

**Figure 1:**
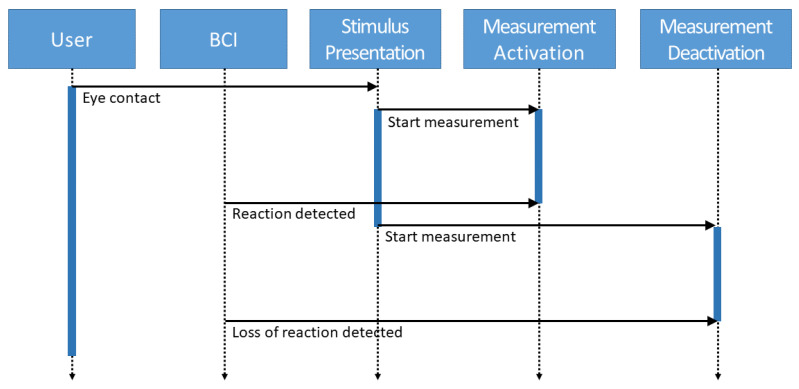
Sequence diagram of measurements for latency calibration.

These latencies are determined as shown in Figure 1. The user first makes eye contact with a visual stimulus by looking at a designated position. The stimulus is then presented there, and the reaction time measurement is started. As soon as the BCI detects the reaction in the visual cortex, the measurement of this latency is stopped. Subsequently, the stimulus is removed, and the measurement of the second latency is started. This is again terminated when the BCI registers at the visual cortex that the evoked potential sufficiently subsides.

In the interaction elements described below, swelling or deswelling values are presented over a period of time. Value input can be achieved by allowing the user control when looking at (or away from) the VEP stimuli. Since the values for the latencies are now known through the calibration step, these can be used for the interaction elements to determine what value was presented at the time currentTime−latency on the screen. Since the accuracy of the user’s input is mainly dependent on the accuracy of the latency determination, this is examined in the experiment (see Section 4).

### 3.3. Interaction Elements

**Figure 2:**
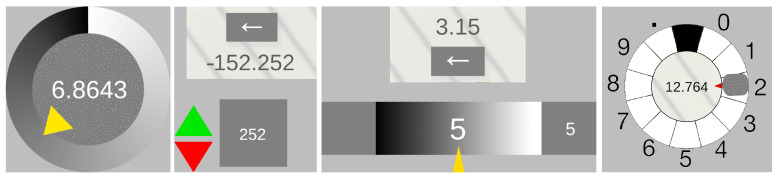
Interaction Elements. Circle, Time Change Controlled, Bar, and Dynamic Circle Controlled Element (from (**left**) to (**right**)).

We propose a set of novel interaction elements for value input through VEP (see Figure 2). For the design of user interfaces, there are a number of guidelines (such as [46,47]). These contain many important hints for ensuring that user interfaces are easy to use. We adopt the principles that the user should be guided by familiar elements and that they should always be aware of the state of the interaction. In this paper, however, we focus on the interaction elements themselves at a more granular level. To this end, we derive some aspects from the design of eye tracking. The advantage of both eye tracking and VEP is that the interaction can be carried out hands-free and only with the eyes. In contrast to eye tracking, however, no light-sensitive camera system is required for tracking the eyes and mapping what is seen. However, some ideas from gaze tracking can be adapted.

For the user interface design, we are guided by classic GUI elements (widgets) and adapt these for VEP-supported control. When selecting the UI elements, circular menus are useful (as in [44]), as the user does not have to move their gaze far to recognize the visual feedback of the interaction. In addition, other familiar interaction elements (see [45]) such as repeat buttons and sliders are used to implement composite widgets. Finally, it is tested whether the Midas touch problem, which has been reported with eye tracking, also presents a difficulty with this interaction technique.

#### 3.3.1. Circle Controlled

The first defined element allows a direct complete value input with only a static stimulus. The value is changed incrementally over the gaze’s dwell time, starting from a minimum value via a value increase up to a defined maximum. Because the value increase is unidirectional in one direction, the value should immediately start again at the minimum after reaching the maximum without needing repeated interaction initialization.

The value range is represented as a ring on which a pointer element similar to a clock circle is located as an indicator of the currently reached value. The visual stimulus is placed in the center of the ring as a filling circle. Centered and in the foreground is the value display in the form of a text field. The value display changes directly proportional to the pointer movement. The rising value can thus be observed until the desired value is reached and defocused when it is reached. We suppose that eye contact is accidentally broken prematurely even though the expected desired value is not yet reached. In this case, the VEP interaction can be continued by refocusing the stimulus, i.e., increasing the value. A value that is inadvertently chosen to be excessively high results in an above zero result in a further round. The advantage of positioning is that the focus is on the value display and not actively on the stimulus, and all information necessary for the value setting is in the focus area. When eye contact is broken, the value is back-calculated based on the latency obtained in the calibration. The interaction element is static and does not perform any independent movement in the scene.

**Figure 3:**
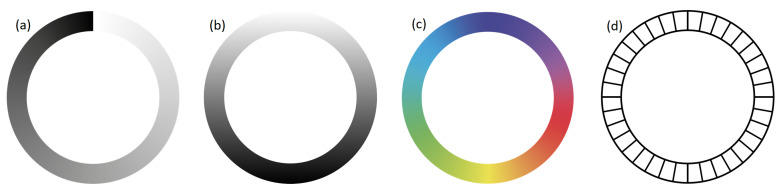
Variations of circle controlled input: (**a**) min to max value, (**b**) continuously increasing and decreasing value, (**c**) association of defined colors/values with transitions, (**d**) discrete value/state changes.

Different input possibilities can be realized by exchanging the associated values on the slice. As described, the values can continuously increase from a defined minimum to a specified maximum (see Figure 3a). This can be used according to a classic slider GUI widget. Since the value in Figure 3a clips from the maximum value to the minimum value at the end of a cycle, a variation of this may be helpful in some applications. Figure 3b shows a continuously increasing value and a continuously decreasing value in the middle. For example, these value curves can be used when a positive or negative difference to a value is to be approximated. A possible application here would be the displacement of 3D objects to a suitable distance to other objects. Another alternative is to associate positions on the circle with multidimensional values (see Figure 3c). An example of such an application would be the well-known circular color picker GUI widget. Finally, this allows us the definition of not only continuous but also discrete value changes (see Figure 3d). Alternatively to the discrete values, of course, a set of interdependent or also independent actions could be executed. This could be used to implement an alphanumeric selection.

#### 3.3.2. Time Change Controlled

This interaction element is also equipped with only one static stimulus, and the value display is also positioned centrally in front of the stimulus. Unlike the Circle Controlled element defined in the previous section, the values can be changed bidirectionally in two directions with only one stimulus. This is achieved using a time-switching mechanism, which is realized by additional detection markers. Thus, values can be corrected directly. For this purpose, there are arrow elements that point upwards to increase the value and downwards to decrease the value. These are placed at a distance to the side of the stimulus so that no accidental stimulus reaction of the visual stimulus takes place. The change in the arrow elements takes place after a fixed time interval.

In addition to this one-dimensional control, further time phases for a dimensional increase in the value change would also be possible, for example, to be able to switch between additional states in addition to intensity. Further detection markers could integrate this into the time schedule to enable any number of states. This time phase forms the action window to change the value in one direction via stimulus activation. Besides the pure settling time, the activation time is added for this interaction technique. If the element is focused shortly before the current time interval expires, the activation time takes longer than the duration of the time window being open. Accordingly, the activation falls into the following (in case of bidirectionality into the opposite) time window. To avoid this problem, the activation time is calculated back from the activation time to detect whether the eye contact has already started at the previous time window. Accordingly, the value input takes place over two successive interactions. In the first interaction step, the sign, as well as the digits before the decimal point, are determined in integers, and in the second step, the digits after the decimal point are determined. When eye contact is interrupted, the value is calculated back, and the direction of the value change is determined by the activation time based on the latency obtained in the calibration.

#### 3.3.3. Bar Controlled

The third interaction element defined here is based on the concept of a classic horizontal GUI slider and consists of two static stimuli. In the center, there is a bar which, similar to the Circle Controlled interaction element, shows a scale for the display of the value interval and a pointer element as an indicator for the percentage of the value reached as a line. For a bidirectional value change, stimuli are placed on the right for value increase and the left for value decrease. The value input is step-wise for each digit. The input values per interaction are thus integers from zero to nine, from which continuous values result in a row. The input possibility lies in the interval of [0; 9] and carries several decimal places. The decimal point is always set automatically after the first entered number. The display of the currently selected digit is placed in the center of the scale and for monitoring above the presently focused visual stimulus. Centered above the scale bar, the already composed continuous value is displayed. An additional visual stimulus is placed below the bar to correct the last entered value. When eye contact is broken, the value is recalculated based on the latency obtained in the calibration. After the interaction is finished, the element’s state is set back to the initial state except for the value entered so far. This element can be used like the classic GUI widget slider or a text field for entering values.

#### 3.3.4. Dynamic Circle Controlled

Unlike the previous elements, the Dynamic Circle Controlled interaction element has a single dynamic stimulus, not a fixed one. For interaction, the stimulus must be tracked in its movement with the eyes after stimulus response detection. The stimulus circles on a ring track which corresponds to the value range, similar to a clock, and serves as an indicator for the currently reached digit. The digits, placed in segments of the value range, are reached incrementally over the dwell time, starting from a minimum via a value increase up to a defined maximum and an additional decimal point to separate the digits before and after the decimal point. As with the circular element, the increase in value is unidirectional in a clockwise direction. If the reaction detection continues, the movement of the stimulus leads beyond the zero point and results in another round. The digits of the segments are positioned so that they are never covered by the stimulus and are always recognizable. In the center of the ring is the value display in the form of a text field. The value display expands directly after each completed interaction, coupled with detecting response loss. The stimulus can thus be observed until the desired character is reached and defocused when it is reached. If eye contact is broken, the value is back-calculated based on the latency obtained in the calibration. The stimulus is subsequently returned to its initial state, similar to a telephone dial. If the eye contact inadvertently breaks prematurely even though the desired value has not yet been reached, the result is an erroneous input. Deletion of the last input can be performed via an additional stimulus attached to the side of the element. The inputs made result in the continuous value when lined up and are displayed in the center of the value range. The value can thus be checked in the element’s center within the interactions. The application options correspond to those of the Circular Controlled elements.

## 4. Experimental Setup

To test the feasibility of our concept and perform a corresponding evaluation, we developed an experimental application that implemented VEP in the context of a desktop as well as a VR environment.

### 4.1. BCI

**Figure 4:**
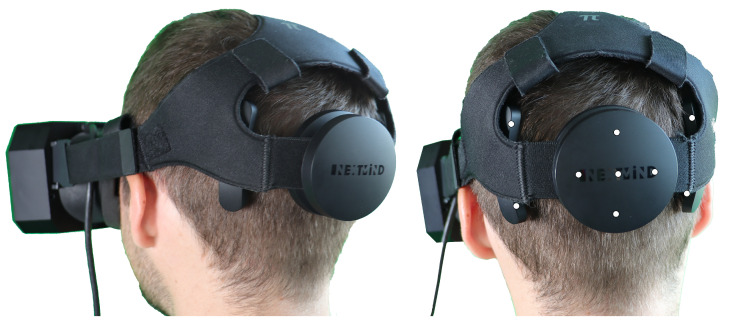
User wearing HMD-*Pimax 5K* and BCI *NextMind*. Electrode positions marked as white dots on the right side.

Due to the requirements for the system raised in Section 3 and the devices used in other approaches (see Table A1), the BCI Development Kit from manufacturer *NextMind* (see Figure 4) was used in the implementation. Despite being available off the shelf as a pretty inexpensive consumer product, it has a high detection and transmission rate according to [21,28]. It has small dimensions, can be used with an HMD for VR applications, and allows a high degree of head movement [48] through a Bluetooth LE connection. There are a number of research papers [21,28,49,50] using this device. However, of these approaches, as already discussed in Section 2, VEPs have up to now been only used as action triggers or buttons.

One advantage is the dry electrodes for recording the EEG signal. For VEP applications, Hemptinne et al. [51] undertook studies on the acuity of VEP and proposed Iz, Oz, POz, O1, PO7, O2 and PO8 as the most sensitive electrode positions of the standard 10-10 system location (see [52]). These positions are where the NextMind device is located. However, according to Zheng et al. [53], in many studies, fewer electrode positions were successfully used such as Oz, O1 and O2.

Research in [54] compared EEG performances between dry and wet electrodes. It concluded that dry electrode headsets are less sensitive to electromagnetic interference that can occur in clinics or at home. This type of headsets falls under the category of CVEP-based BCIs and thus allows interactions with a high selection speed [55,56].

Proprietary signal processing is performed directly after the encrypted brainwave data are received by the *NextMind Engine*. For this, machine learning algorithms are used to decode and recognize brain activity and active visual focus in real time. On the negative side, however, it does not grant an open interface to the raw data. However, the API provides a confidence value to each registered stimulus, making it possible to determine the degree of confidence with which a particular stimulus was recognized.

The *NextMind*-BCI supports a maximum of ten simultaneously active stimuli [28], which is sufficient for our requirements. Here, the flashing of the stimulus is represented by a microbe-like structure which is applied to a gray background without being visually prominent. This texture covers only a part of the background. NextMind recommends 4.3${}^{\circ }$ of the gaze center as the stimulus size we use in the experiment. The temporal change between the faded-in and faded-out stimulus structure leads to the stimulus-emitting state, which the BCI detects as evoked potential.

### 4.2. Test Environment

We used a desktop computer with a 27-inch display with 4k resolution. As VR goggles, we used a Pimax 5K with an HTC base station, allowing the simultaneous attachment of the NextMind BCI to the back of the head. The NextMind BCI can only be used within the Unity LTS runtime environment. To enable virtual reality functionality in the application, the Unity plug-in SteamVR communicating on the OpenXR interface was integrated. For the following experiments, a scene was modeled to act as an environment for the integrated interaction elements. Action logging functions were implemented in this, which wrote the user interactions and all internal processes such as states, calculations, or errors to a log file for later evaluation.

### 4.3. Implementation of Interaction Elements

The interaction elements were implemented as shown in Figure 2. After the system detects a visually evoked potential of a stimulus, acoustic feedback is given to the user in the form of a beep. The exact implementation of the user study is described in the following section.

## 5. Evaluation

We conducted a user study that consisted of two parts. In the first part, we wanted to evaluate our hypotheses for the calibration, which forms the basis for our proposed interaction technique:

- $H_{a}$: The calibration time varies depending on the number of stimuli;
- $H_{b}$: There is a difference in calibration time between a desktop and a VR setting;
- $H_{c}$: VEP reaction and settling time differ;
- $H_{d}$: Reaction and settling time differ between all users;
- $H_{e}$: Timings are stable for all users.

In the second part, we evaluated whether the participants could use our proposed interaction elements and obtain qualitative feedback for improvements.

### 5.1. Methods

#### 5.1.1. Participants

Subjects from the extended university environment were randomly asked if they wanted to participate. A total of 10 volunteers participated in our study, including 5 females, aged between 25 and 32 years (mean age 28). The participants were invited individually, spread over three test days. Written informed consent was obtained from all participants after they were informed about the goal of the study. Of these, five confirmed usually wearing glasses for vision correction whilst four wore their glasses during the evaluation. Participation was not restricted to a target group in terms of age or gender. Still, no evaluation was performed on individuals with photosensitive epilepsy, as this posed too high a risk of seizures from the visual stimuli of the VEP. None of the participating individuals had experience with either VEP or brain–computer interfaces. All participating persons already had experience with VR using an HMD. During the study, the information required for the evaluation was stored anonymously so that it could not be traced back to the participating persons. None of the participating persons showed BCI deficiency.

#### 5.1.2. Apparatus and Materials

We used the hardware and software system defined in Section 4. The HMD was spatially calibrated again at the beginning of each evaluation day. The screen, as well as the HMD, were set to a constant refreshing rate of 90 Hz. To optimize the response in the visual cortex, the screen brightness was set to maximum at the beginning of each evaluation. Light reflections on the screen were reduced by rotating the screen away from light sources. In addition, the chair and screen height were adjusted individually for each participant. During the desktop test, each participant sat about 50 cm away from the screen. The test site was located in a quiet open-plan office.

For the input of continuous values, values were first determined which were evenly distributed on the respective scales of the interaction elements. Very small values with low digits required fast input for Circle and Dynamic Circle Controlled. Very large values, in turn, required longer viewing times for visual stimuli. To test the time-switching phases for the Time Change Controlled element, positive as well as negative values were added. However, due to the paired input, maximum values up to ‘23’ were chosen for this. For the bar controlled Element, special care was taken to evaluate digit ‘5’ due to the start position as well as the respective ends of the scale, ‘9’ and ‘0’. The repeated input of digits, as well as the choice of the most distant values, ‘0’ and ‘9’, or ‘,’ was observed.

#### 5.1.3. Procedure

At the beginning of the study, each participant received a verbal explanation of the background, the aim, procedure, and instructions for use. In addition, a briefing on the modes of operation and interaction steps of the individual elements was provided before implementation. Informed consent was obtained from all subjects involved in the study.

##### Preparation

To allow the best possible connection of the EEG electrodes on the scalp, the BCI had to be placed on the back of the head (see Figure 4) in the area of the visual cortex [57]. The reference point was the inion, i.e., a small bump at the back of the skull, which can be easily felt by hand by the subjects. The hair could be parted at the contact points by slightly moving the sensor up and down, which was previously fixed via the headband. This enabled the best possible direct contact between electrodes and the scalp. In order to guarantee an optimal and stable connection during use, care was taken to ensure that the BCI was fixed tightly enough but not uncomfortably. This prevented the sensor from becoming dislodged during slight head movements and interrupting signal transmission. For use in a desktop mode, the supplied headband was used. Using the HMD, the BCI was attached to the back of the VR’s headband.

##### Calibration of BCI Device

During calibration, all EEG electrodes had to be in direct contact with the scalp. Connection quality was assessed (see Table 1) by viewing the contact status during the running application via the NextMind Profiler software tool belonging to the BCI. This indicated the connection quality by means of a scale with descending values from A to D. The quality of connection was evaluated by means of NextMind Profiler.

The evaluation run was continued for all subjects only when a calibration result of at least Level C was achieved. Due to the reduced pressure that the headband of the VR goggles could exert on the back of the head and the hair, the achievable connection quality was limited (Table 1). For three subjects, sufficient contact could not be established in the VR setting. Therefore, they were not considered in the evaluation of mean latencies.

##### Sequence of Evaluation

**Figure 5:**
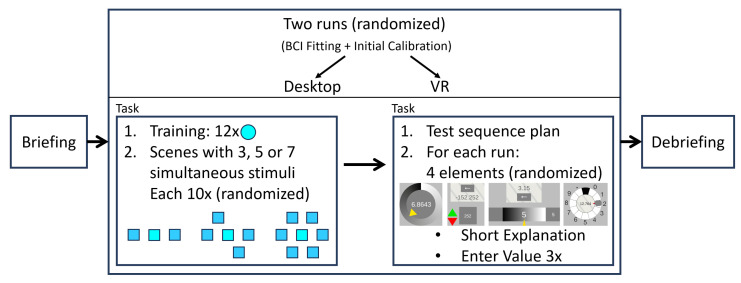
Sequence of Evaluation.

After briefing a subject as described in the beginning of this section, a run with either the desktop setting or the VR setting was performed first on a randomized basis (see Figure 5). Preparation and calibration of the BCI device were performed as described using the supplied headband (desktop setting) or VR headbands (VR setting). The study was carried out by way of two successive tasks. First, determination of the latency value was conducted; then, evaluation of the value input by the VEP interaction controls was performed.

In the first task, a training phase was carried out. The participants were instructed to permanently fixate the turquoise sphere in the center of the screen with their eyes. The VEP stimulus was faded in and out. The times of the potential detected by the BCI were recorded by the application. This procedure was repeated 12 times. Subsequently, subjects were randomly presented with alternating scenes of 3, 5, and 7 simultaneous VEP stimuli with instructions to fixate the stimulus located in the center. The measured latencies were recorded by the application. In total, the scenes of 3, 5 and 7 stimuli were presented 10 times each.

In the second task, a test sequence plan was first set up. First, the four VEP interaction elements were verbally explained to the participants, who were given the opportunity to test the value input with these elements. For the Circle Controlled Element and the Time Change Controlled Element, the range of values included [−9.99; 9.99] and two decimal places. For the Bar-controlled and Dynamic Circle Element, numbers had to be entered digit by digit with five digits and a decimal point. The value display with the underlying stimulus had to be focused to activate the value change. When the given value was reached, the eyes had to be closed to accept the value or digit and end the interaction. Subsequently, the participants were shown the elements randomized with the instruction to enter a defined value by means of the respective interaction element. Before entering a new value, the VEP interaction elements were reset to their original state. In total, each of the view interaction elements was presented three times. The order of the presented interaction elements was randomized, but remained in the same order for both runs (desktop/VR).

During the experiment, subjects were instructed to spontaneously express their thoughts aloud so the experimenter could record them. Finally, at the end of the experiment, qualitative feedback was collected using a brief questionnaire.

### 5.2. Results

The technical setup, including the BCI, worked without problems for each session. However, in the VR setting, only 7 of 10 subjects could participate because the headband of the HMD using the BCI could not exert enough pressure on the EEG electrodes through long and voluminous hair (similar to what is stated in [28]). The step with the calibration could also be performed as planned. However, the step with the value entries (both desktop and VR execution) was frequently interrupted and aborted during the value entry due to premature detection of the loss of response. Accordingly, some value inputs had to be performed in multiple steps. Accidental activation of the interaction was triggered several times, as these interactions took up a relatively large area on the screen and attracted the user’s attention. In all runs performed (desktop, VR), all three input tasks of the four interaction elements were processed.

**Latency.** The data could be aggregated and analyzed based on the logs recorded during the test. For hypothesis $H_{a}$, there was no significant difference between the different numbers of stimuli activated simultaneously. An ANOVA evaluation showed that this was not the case for either the reaction or the settling time condition: F(2, 27) = 0.40, *p* = 0.67. According to hypothesis $H_{b}$, no difference in calibration time between the desktop and VR settings could be determined. No significant difference could be detected in response t(15) = −1.11, *p* = 0.28 nor in settling time t(15) = 0.63, *p* = 0.54. For hypothesis $H_{c}$, a difference was observed between the duration of response and settling time, but it was not significant: t(58) = −1.79, *p* = 0.08.

For $H_{d}$, i.e., the consideration of whether there were significant differences in the reaction and settling time between all users, a relatively high standard deviation (desktop: $\bar{x}=1.915\pm 0.293$, VR: $\bar{x}=2.173\pm 1.016$) for reaction time was detected, but a low standard deviation (desktop: $\bar{x}=2.063\pm 0.097$, VR: $\bar{x}=2.049\pm 0.007$) for settling time was found (see Table A3, Appendix B)).Finally, for hypothesis $H_{e}$, the measurement data showed that the standard deviations in the test performance of the individual users were relatively high for activation time but extremely low for settlement time. On average, the standard deviations for activation time (desktop: σ=1.036, VR: σ=4.836) and settlement time (desktop: σ=0.001, VR: σ=0.001) were determined.

**Value input.** The results of the value inputs for the individual controls are listed in Table 2. The average distance between the intended and actual value inputs was calculated for the continuous value inputs of the Circle Control and the Time Change Control. Since Bar Control and Dynamic Circle Control allow an exact input, the Hamming distance was chosen to measure the deviation of the intended input, which reflects deviations in the single decimal digits between the intended and actual value inputs. Table 2 also shows the average value increase time of the different controls.

### 5.3. Discussion

To evaluate the latency correction and to examine the value inputs of the proposed interaction elements for their accuracy and usability for continuous value input, a user study was conducted with ten subjects. This section discusses the latency determination results and value inputs using the proposed VEP widgets.

**Latency.** From the results of Section 5.2 on latency determination, it can be concluded that the reaction time is too inaccurate to perform an interaction with it successfully. However, on the other hand, this also shows that the settling time has a very stable value over a very stable value for back-calculating the set value. It is stable not only for a single user but also over multiple users ($H_{d}$ and $H_{e}$). The evaluation shows no dependence between the number of simultaneous stimuli and the settling time ($H_{a}$). When minimum distances are maintained, this can be used to create complex VEP interaction elements that involve multiple stimuli for control. No significant difference is shown between the desktop and VR settings. The difference is ($H_{b}$); thus, this latency is suitable for both display types.

**Value Input.** When the error distances, which define the accuracy of value input per digit, of Table 2 of the individual interaction elements are compared with each other, the interaction within the VR application of all elements has a higher average error. Thus, using BCI via desktop leads to more accurate input of values than those within VR.The results show an average deviation of 0.53 for the bar controlled interaction element in the desktop run and 0.75 in the VR run around the specified value. From this, it can be concluded that the free value input with one digit per interaction step achieves the best accuracy.

If the results from Table 2 are considered, it can be seen that under the aspect of the Hamming distance per digit, the bar controlled element has the best result with a value of 0.42 in the desktop run and 0.53 in the VR run (see Row 1). Equally, however, this shows that this implementation of the interaction techniques often does not allow for accurate input but only approximate input.

Comparing the average value increase time of the interaction elements (see Line 3), it can be seen that the bar controlled element achieves the best results with 2.29 seconds in the desktop and 2.41 in the VR run. The reasons for this are the direct selection of the value change direction due to the use of two stimuli, as well as the starting value ‘5’ placed in the middle of the scale. The problem and reason for the increased value increase result of the Dynamic Circle Controlled interaction element is the stimulus, which is challenging to focus and track, according to the participating subjects of the user study. The movement of the stimulus leads to premature interaction terminations, which have to be resumed. In the case of the Circle Controlled interaction element, the rapid numerical update of the visual value display in the tenths and hundredths of a second range is shown to cause stimulus overlap and thus to superimpose the VEP stimuli. The evoking factor is influenced to such an extent that, on average, three terminations occur within one value input. Increased values in the Time Changed Controlled interaction element result from waiting times for the required phase, which switches only after a time interval.

If the interaction technique is compared with work [25], in contrast, it can be determined that the using person does not have to make any own adjustment to the system. When controlling the avatar, a redirection has to be initiated several movement steps before the planned change in direction. With the latency correction, this is no longer necessary. The predefined decimal place values of the Time Change Controlled interaction element are in the two-digit range and require an equally long focusing of the stimulus to reach them. Compared to the pre-decimal digits, which are in the single-digit range, only about a third of these are achieved. From this, it can be concluded that the longer a visual stimulus has to be focused, the higher the reaction loss probability that the interaction is terminated prematurely. Therefore, value inputs that go beyond the input of a one-digit value, as in the case of direct or partial input, are unsuitable.

The Midas touch problem is not prominent with the VEP widgets since, due to the latency, the values and states can be looked at for a sufficiently long state (∼2 s) without initiating a VEP interaction. Especially for experienced users, this is not a significant problem.

## 6. Discussion and Limitations

The participants of the study easily understood the functionality of the VEP interaction elements and were able to use them. However, the most challenging part for them was to find the right moment to close their eyes in order to set the value. Here, the input accuracy depended significantly on the temporal change in the values in the interaction elements. The faster the values changed, the more difficult it was to hit the exact value. Hence, the value input by VEP interaction elements faced a trade-off of the value range, the desired accuracy of the value input, and the time available to perform the input. Due to the fact that the values change (increment) continuously, it was possible for users to estimate the value in another specific time interval. We hypothesize that it is possible to train this temporal targeting of the desired value through practice, thereby further improving input accuracy. We will address this assumption in detail in a further study.

Our approach was successfully evaluated with consumer hardware, which is easy to use (dry electrodes and fast setup) and relatively inexpensive (dissemination). However, as shown in the calibration process (see “Calibration of BCI Device” in Section 5.1.3), this hardware is limited in terms of signal quality. Especially for people with long and thick hair. However, in contrast to the device used, there are BCIs that have sensors with longer prongs that provide good signal quality even with more voluminous hair. The signal quality also differed significantly between desktop and VR settings. A correlation may exist between the performance of the value input and the signal quality. Other influencing factors could be a different resolution as well as contrasts between the output technologies. In subsequent studies, we will investigate these continuative hypotheses with reference devices in order to determine whether these differences had a significant impact on performance.

In this paper, we first introduced the foundations of temporal control by VEP. We then evaluated the feasibility of our proposed VEP interaction elements (widgets). However, the constraints imposed by the laboratory setting hindered the investigation of the practical applicability of our approach. In subsequent work, we will investigate the applicability through different existing applications. To accomplish this, GUI widgets should be replaced with VEP widgets in well-known graphical application programs. Thus, the substitutive versus complementary use of VEP widgets versus GUI widgets can be analyzed in an applied context.

Another interesting question could be the following: How can temporal control be applied to known areas of application of VEP? A future study could investigate the ways in which it would apply to the input of letters. As described in Related Work section (see Section 2.2), this often requires multiple input steps or the display of many simultaneous VEP stimuli. With an appropriate VEP widget, the letters of the alphabet could be displayed in ascending order over time. The moment of gazing away from the widget would enter the last seen letter.

We tested our concept in VR and on desktop. However, there are also approaches (see Appendix A) that render the VEP stimuli via LED. This use of VEP, independent of complex displays, allows for more diverse integration into human–computer interfaces. In contrast to existing approaches, it is now conceivable to apply it to real-world environments, where the user can not only turn things on and off by looking at VEP stimuli (such as a light switch), but also gradually adjust a certain brightness by temporal control. In our further studies, we are working on this transfer of our concept to real environments. In a real-world context, however, a variety of other influencing factors will have to be taken into account. For example, the ambient brightness, interfering natural and artificial light sources, the ideal placement of the stimuli, or the simultaneous control of objects by multiple users will have to be examined.

## 7. Conclusions

BCIs are becoming more convenient to use and have recently reached the mass market. They also have various applications, including those for visual evoked potentials. However, previous approaches used VEP only as action triggers or switches. This paper described the observation that the latency of the loss of the visual evoked potential is a reliable temporal parameter. This parameter can thus be used to create new manifold forms of interaction. Based on this latency, we introduced the novel concept of temporal control for VEP. By looking at a stimulus, an interaction element is activated and changed over time, until the user averts their gaze. This allows the user control of the extent of the change. We used this concept to develop VEP widgets that allow control of time-varying variables by looking at them. In contrast to previous approaches, with such widgets, VEPs can be used directly for value input.

Our user study showed that the latency for the loss of the VEP was very stable for each subject and also between subjects. Similarly, in our study, we tested the proposed VEP widgets for both a desktop and a VR setting. In both cases, the feasibility of these concepts was demonstrated. Based on the results of the study, we discussed the successes and limitations. This allowed us the development of a number of further hypotheses and ideas (see Section 6). Our approach even works with inexpensive, mobile devices that are commercially available, which supports its dissemination.

Having introduced the concept of timed VEP widgets in this paper and demonstrated their feasibility, our future research will extend to optimization and to other application areas. To this end, we will conduct a review through an in-depth, larger scale study of influencing parameters such as signal quality on performance through reference devices. We will also investigate training effects on temporal control, in particular whether timed looking away from the VEP stimulus can be performed more accurately through practice. As described in Section 6, our future work will focus on using VEP widgets as replacements or as complements to GUI widgets in known applications in order to obtain better evidence of their external validity. Finally, future research will be focused on transferring our concept into real-world applications by using our concept in smart environments.

## Figures and Tables

**Table 1:** *NextMind*-BCI calibration results.

	Distribution			
**Signal quality**	**A**	**B**	**C**	**D**
**Desktop**	3	7	0	0
**VR**	0	5	2	1

**Table 2:** Results of value inputs by VEP interaction elements.

	Circle		Time Change		Bar		Dynamic Circle	
	**Control**		**Control**		**Control**		**Control**	
**Pass**	**Desk.**	**VR**	**Desk.**	**VR**	**Desk.**	**VR**	**Desk.**	**VR**
Mean error	2.44	2.38	1.19	1.83	-	-	-	-
Mean hamming dist.	-	-	-	-	0.42	0.53	0.68	0.67
Input time	3.34	4.45	2.46	2.62	2.29	2.41	4.56	6.88

## Data Availability

Data are contained within the article.

## Appendix A

The tables in this appendix are adapted versions from our short paper [15].

**Table A1:** Overview of categorized related work about pure VEP approaches.

Ref.	Used	Presentation	Quality of	Number	Application
	**Hardware**		**Classification**	**of Stimuli**	
**Navigation task, static stimulus**					
[58]	Olimex	LED	≈60%	4	Cursor
[59]	ADInstruments BioAmp	LED	93.14% ± 5.73%	8	Cursor
[22]	BioSemi ActiveTwo	DESKTOP	—	4	Game/2D
[25]	Emotiv Epoc X	DESKTOP	80.37% ± 11.85%	4	Game/2D
[60]	Emotiv Neuraheadset	DESKTOP	—	4	Game/2D
[61]	NI DAQ-Pad 6015	LED	65–100%	4	Game/2D
[23]	—	DESKTOP	—	4	Game/3D
[28]	NextMind	VR	99–100%	6	Game/3D
[26]	g.tec-g.USBamp	DESKTOP	≈97.14%	4	Robotics
[62]	MindMedia	DESKTOP	≈91.75%	4	Robotics
[63]	—	LED	≈87%	48	Robotics
[32]	Emotiv EPOC+	AR	≈83.3%	4	Robotics
[64]	—	AR	≈89%	4	Robotics
[11]	—	DESKTOP	93.53% ± 5.18%	2	Traffic
[65]	BioSemi ActiveTwo	DESKTOP & VR	89.6% ± 11.3%	4	Game/3D
[27]	g.tec-g.USBamp	DESKTOP & VR	≈98.20%	3	Game/3D
[66]	Emotiv EPOC+	VR	≈62.54%	4	Keyboard
**Navigation task, dynamic stimulus**					
[48]	—	DESKTOP	—	4	Cursor
[24]	Biosemi EEG + Neurscan	DESKTOP	≈94.75%	4	Game/2D
[12]	g.tec - g.MOBIlab+	VR	—	4	Game/3D
[31]	Ultracortex	LED	≈79.4%	4	Robotics
**Value input task, static stimulus**					
[67]	Neuracle Wireless	AR	≈83.85%	6	Keyboard
[68]	OpenBCI Ganglion	VR	100%	4	RemoteCtrl
[69]	Neuracle Wireless	AR	93.96% ± 5.05%	4	Robotics
[70]	BrainAmp/AntiCamp	AR	≈30.51%	6	SmartHome
[33]	g.tec-g.USBamp	DESKTOP	—	5	Keyboard
[37]	g.tec	DESKTOP	≈92.25%	5	Keyboard
[34]	—	DESKTOP	—	13	Keyboard
[36]	Neuroscan EEG	DESKTOP	≈95.8%	36 & 6	Keyboard
[71]	g.tec-g.USBamp	DESKTOP	≈96.17%	4	Keyboard
**Value input task, dynamic stimulus**					
[29]	—	VR	—	—	Game/3D
[30]	OpenBCI Ganglion	VR	≈83%	—	Game/3D
[72]	—	LED	≈83.26%	—	SmartHome
[73]	g.tec Nautilus	AR	≈89.3%	4	SmartHome

**Table A2:** Overview of categorized related work about hybrid VEP approaches.

Ref.	Hybrid	Used	Presentation	Quality of	Number	Application
	**Extension**	**Hardware**		**Classification**	**of Stimuli**	
**Navigation task, static stimulus**						
[74]	MI	—	DESKTOP	—	2	Cursor
[75]	P300	Emotiv EPOC+	LED	—	4	Robotics
**Value input task, static stimulus**						
[21]	Passive	NextMind	DESKTOP	100%	4	Keyboard
[17]	GAZE	Neuroscan Synamps2	VR	≈97.5%	40	Keyboard
[19]	EMG	Neuroscan	DESKTOP	80.8% ± 15.6%	4	Keyboard
[76]	P300	BrainProducts actiCHamp	AR	≈85.70%	4	SmartHome
[77]	P300	g.tec-g.USBamp	DESKTOP	100%	—	SmartHome
[16]	GAZE	Emotiv Neuraheadset	AR	70.94% ± 1.29%	3	RemoteCtrl
[78]	GAZE	g.tec-g.MOBIlab+	DESKTOP	—	2	SmartHome
[18]	EOG	BioSemi ActiveTwo	VR	97.74% ± 3.89%	9	Keyboard

## Appendix B

**Table A3:** Mean latencies (in s) of participating persons *i*.

			Mean Latencies of the Participating Persons											
**Device**	**Phase**		**1**	**2**	**3**	**4**	**5**	**6**	**7**	**8**	**9**	**10**	$\bar{x}$	σ
Desktop	Reaction	${\bar{x}}_{i}$	1.8715	2.2607	1.7426	1.9937	1.5778	1.7405	2.5553	1.9844	1.9549	1.4727	1.9154	0.2930
		$\sigma_{i}$	0.1976	0.5693	0.2729	0.8815	1.0924	0.3721	4.0659	0.9557	1.3617	0.5926	1.0362	
	Settling time	${\bar{x}}_{i}$	2.0470	2.0397	2.0523	2.0425	2.2283	2.0483	2.0481	2.0412	2.0344	2.0505	2.0632	0.0970
		$\sigma_{i}$	0.0000	0.0003	0.0007	0.0015	0.0019	0.0011	0.0008	0.0011	0.0005	0.0011	0.0009	
VR	Reaction	${\bar{x}}_{i}$	1.9973	2.2517	2.0140	1.4592	2.1214	3.4908	1.8784				2.1733	1.0158
		$\sigma_{i}$	0.1075	0.3600	0.3943	0.3599	10.9283	20.5517	1.1528				4.8364	
	Settling time	${\bar{x}}_{i}$	2.0535	2.0505	2.0404	2.0546	2.0485	2.0504	2.0465				2.0492	0.0071
		$\sigma_{i}$	0.0008	0.0008	0.0003	0.0005	0.0005	0.0011	0.0011				0.0007	
